# Cardanol-Based Materials as Natural Precursors for Olefin Metathesis

**DOI:** 10.3390/molecules16086871

**Published:** 2011-08-11

**Authors:** Giuseppe Vasapollo, Giuseppe Mele, Roberta Del Sole

**Affiliations:** Department of Engineering for Innovation, University of Salento, Arnesano Street, Lecce 73100, Italy

**Keywords:** Grubbs catalysts, metathesis, pentadecylphenol, cardanol, porphyrins, metallo-porphyrins, fullerenes, fulleropyrrolidines

## Abstract

Cardanol is a renewable, low cost natural material, widely available as a by-product of the cashew industry. It is a mixture of 3-*n*-pentadecylphenol, 3-(pentadeca-8-enyl)phenol, 3-(pentadeca-8,11-dienyl)phenol and 3-(pentadeca-8,11,14-trienyl)phenol. Olefin metathesis (OM) reaction on cardanol is an important class of reactions that allows for the synthesis of new olefins that are sometime impossible to prepare via other methods. The application of this natural and renewable material to both academic and industrial research will be discussed.

## 1. Introduction

In recent years, among other natural compounds, the use of cardanol to form various new compounds and hybrid compounds has attracted the attention of scientists [1,2,3,4,5,6,7,8,9,10,11,12,13,14,15,16,17]. Cardanol is a renewable and inexpensive organic natural resource that is easily obtained via the vacuum distillation of roasted cashew nut shell liquid (CNSL) obtained from the spongy mesocarp of cashew nut shells.

**Figure 1:**
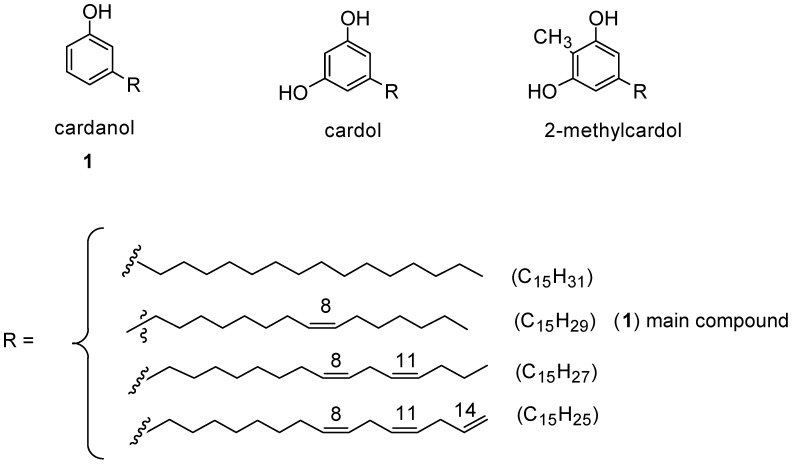
Constituents of technical-grade cashew nut shell liquid.

CNSL is a mixture of cardanol, cardol and 2-methylcardol. All these compounds possess a characteristic long alkyl chain in the *meta* position [1,2,3,4]. Cardanol is the main component (about 84%) of CNSL and is itself a mixture of 3-*n*-pentadecylphenol, 3-(-pentadeca-8-enyl)phenol, 3-(pentadeca-8,11-dienyl)phenol and 3-(pentadeca-8,11,14-trienyl)phenol (Figure 1). Depending on the purification-distillation of cardanol, the monoolefinic 3-(-pentadeca-8-enyl)phenol, can be the main component, accounting for almost 95% on the total; hence, in this work, the term cardanol refers to this monoolefin.

The preparation of fine chemicals from natural and renewable materials has become an attractive topic of research, particularly when the purpose is to recycle the huge amount of agro-industrial wastes to produce, through environmentally sustainable processes, fine chemicals which can be used for different purposes. The extraction of fine chemicals from wastes, such as cardanol, is an old concept. In fact, the phrase “from waste to value”, which means making useful chemical products using the wastes of industry is a well known one. Further, cardanol has a special and unique characteristic: It contains in the *meta* position of the phenolic ring a long alkyl chain that confers attractive properties to cardanol derivatives such as good processability and high solubility in organic solvents; but, also the possibility to influence many chemical transformation introducing novel functionalities [3,4,5,6,7,8,9,10,11,12,13,14].

This paper presents an overview of the developments in olefin metathesis involving cardanol or cardanol-derived molecules for the preparation of new fine chemicals as well new hybrid functional materials, such as porphyrins, phthalocyanines and fullerenes.

## 2. Cardanol: Description and Oil Characteristic

**Figure 2:**
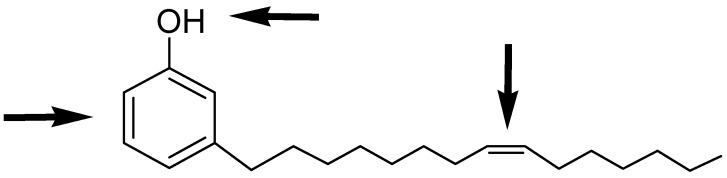
Possible sites for functionalization of the cardanol molecule.

Cardanol is easily obtained by vacuum distillation of CNSL, which is a by-product of the cashew industry. Commercially available CNSL contains a mixture of cardanol (**1**), cardol and 2-methylcardol (Figure 1) in approximately 84, 11 and 5%, respectively. As reported in the literature, by further re-distillation of the liquid, it is possible to obtain a mixture that is rich (90% on average) in the monoolefinic and diolefinic components, together with minor amounts of the triolefin and 3-*n*-pentadecylphenol. The average composition of the mixture is: 20%–30% 3-*n*-pentadecyl phenol, 70%–80% 3-(pentadeca-8-enyl) phenol (**1**), nearly 5% 3-(pentadeca-8,11-dienyl) phenol and less than 5% 3-(pentadeca-8,11,14-trienyl) phenol. *Cis* and *trans* isomers of each component are present in the mixture, but usually the *cis* component is the major one. This mixture can be further purified by successive re-distillation/chromatography. In this way, the monoolefin component, 3-(penta-deca-8-enyl)phenol was obtained almost pure. For simplicity, this isolate will hereon be referred to as “cardanol” in this work. Several types of chemical reactions can be carried out involving the benzene ring, the olefin or the hydroxyl group (Figure 2).

The presence olefin group in the long chain attached to the *meta* position of the phenolic ring allows for the possibility for generating new cardanol-based compounds *via* metathesis reactions.

## 3. Preparation of Cardanol-Based Derivatives

**Scheme 1:**
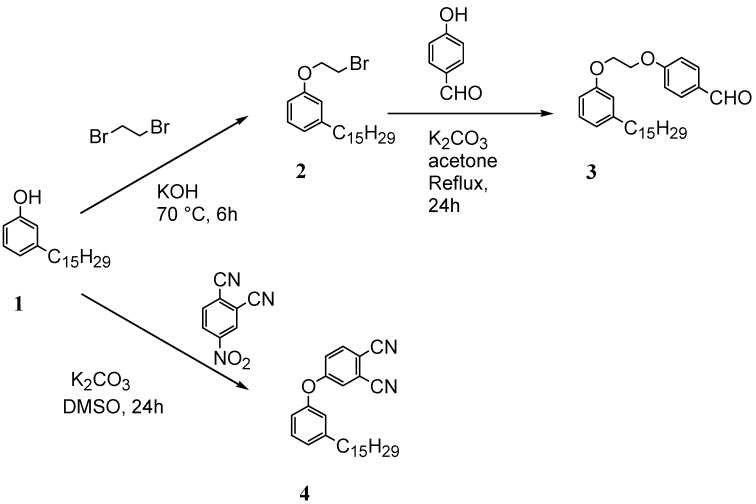
Molecular structure and synthesis of the cardanol-based precursors **1–4** used for metathesis reactions.

**Scheme 2:**
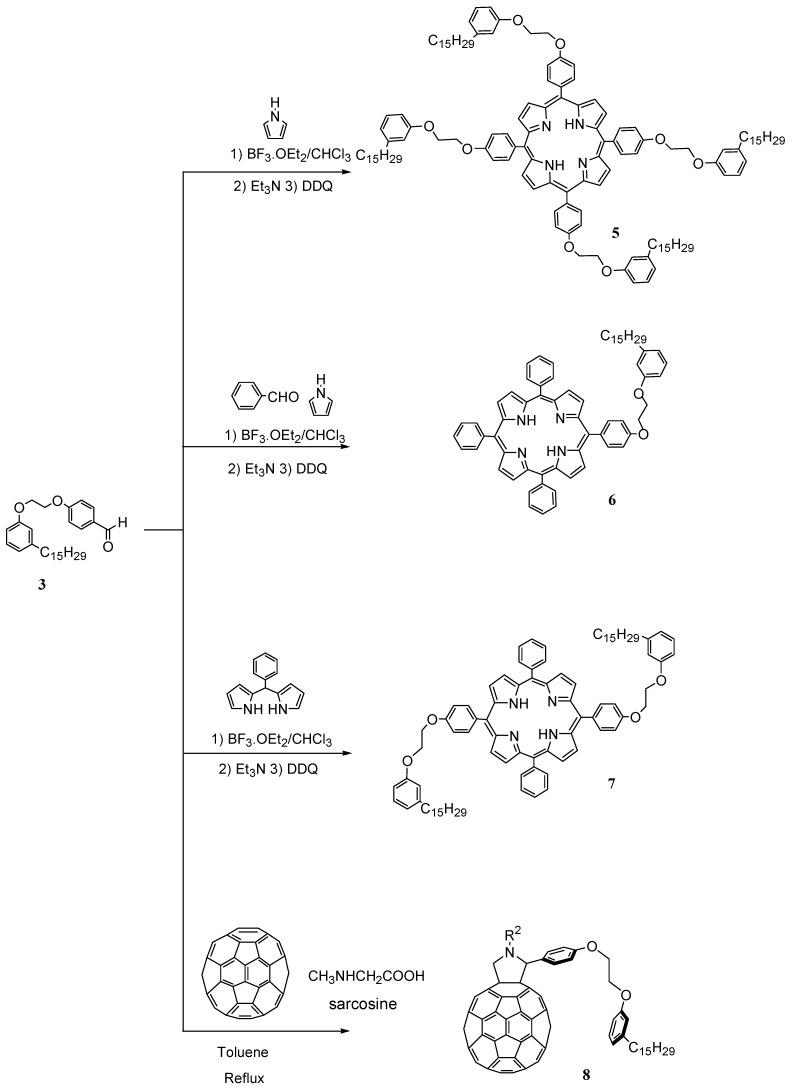
Synthesis of the cardanol-based porphyrins **5–7** and fulleropyrrolidine **8** used for metathesis reactions.

Scheme 1 and Scheme 2 depict the syntheses of several cardanol-based derivatives that have been used as precursors for metathesis reactions.

As summarized in Scheme 1, the compound **2** was prepared via the reaction of cardanol (**1**) with 1,2-dibromoethane in the presence of KOH at 70 °C for 6 h and isolated in 70% yield [15]. Successively, **2** was reacted with 4-hydroxybenzaldehyde in the presence of anhydrous potassium carbonate in acetone under reflux for 30 h to afford **3** in 40% isolated yield. The compound **4** was prepared in 90% isolated yield by reacting **1** with 1,2-dicyano-4-nitrobenzene in DMSO in the presence of K_2_CO_3_ at room temperature for 24 h [16].

As shown in the Scheme 2, 4-[2-(3-(pentadeca-8-enyl) phenoxy)-ethoxy]benzaldehyde **3** was reacted with pyrrole, benzaldehyde or fullerene according to the methods reported in the literature [3,15] in order to obtain cardanol-based porphyrins **5–7** and cardanol-based fulleropyrolidine **8** which can be used as starting materials for metathesis reactions.

In particular, cardanol-based porphyrin **5** was obtained by the reaction of **3** with pyrrole [15]; porphyrin **6** was synthesized by the acid-catalyzed condensation of **3** with pyrrole and benzaldehyde or with meso-phenyldipyrrolmethane [3].

The fulleropyrrolidine cardanol 8 was prepared by the cycloaddition of sarcosine (*N*-methylglycine) in the presence of 3 and C_60_ in refluxing toluene under nitrogen atmosphere for 24 h [17].

## 4. Metathesis on Cardanol and Its Hybrid Derivatives

**Figure 3:**
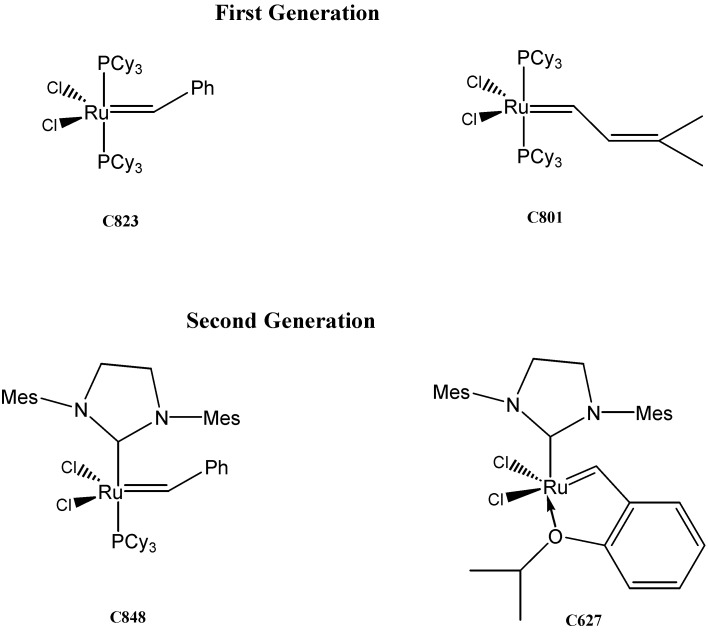
First- and second- generation of ruthenium-catalysts used for metathesis reactions.

As mentioned previously, the olefin metathesis reaction is an organic reaction which offers the possibility to form new olefins [18,19,20,21]. Different commercially available ruthenium-catalysts, which are illustrated in Figure 3 and labelled according to their molecular weights (**C627**, **C823**, **C801**, **C848**), have been used.

**Scheme 3:**
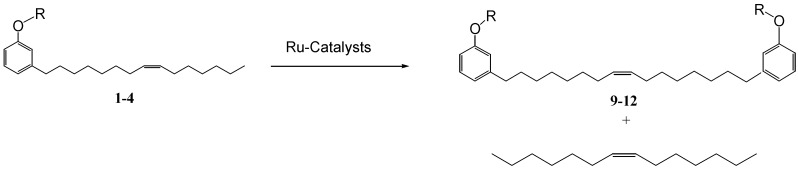
Metathesis reaction involving cardanol **1** and its derivatives **2–4**.

Initially, the mono-olefinic cardanol, 3-(pentadeca-8-enyl) phenol, was used as the starting material to prepare, using a Hoveyda-Grubbs’ catalyst (Scheme 3) [22].

The metathesis reaction was then performed with other cardanol-based substrates to prepare the new derivatives listed in Table 1. The best metathesis conditions resulted used 2 mmol of **1**, and 5 mol% of catalyst **C627** in dichloromethane (1.2 mL) at 40 °C for 45–87 h under N_2_ atmosphere.

**Table 1:** Metathesis performed on cardanol **1** and cardanol derivatives **2–4**.

Substrate	Product	Time (h)	Conv. (%)	Yield (%)
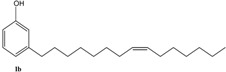	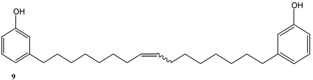	72	78	36
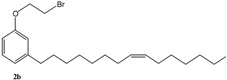	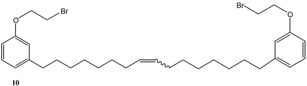	64	86	51
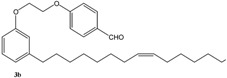	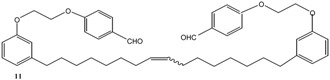	92	62	20
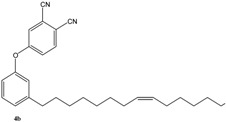	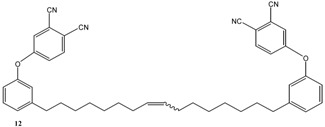	87	55	36

*Reaction conditions*: Starting material (1.0 mmol), catalyst **C627** (2 mol %); dichloromethane (1.67 M). The reaction mixture was refluxed under nitrogen atmosphere.

In all cases, the conversion of **1–4** into **9–12** reached only 42%–65% (isolated yields). This was attributed to the reaction’s reversibility [20,23,24]. All products were formed as a mixture of *Z*- and *E*-isomers, in which *Z*-isomers were dominant according with the results reported by Meek *et al.* [25].

Subsequently, metathesis reactions were applied on cardanol in the presence of commercial olefins bearing –COOH and –COOEt moieties in order to prepare new cardanol-based carboxylic acid- (**15**) and ester-type (**16**) derivatives (Table 2) [26,27].

**Table 2:** Cross-metathesis reaction involving cardanol.

Substrate	Olefin	Product	Yield (%)
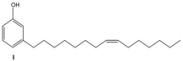	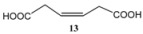	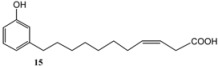	50
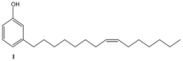	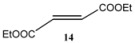	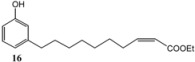	30

*Reaction conditions*: Substrate 1 (3.0 mmol), substrate **13 (**or **14)** (3.0 mmol), **C627** catalyst (5 mol%); dichloromethane (15 mL). The reaction mixture was stirred under reflux in an oil bath.

In 2006, the preparation and characterization of new hybrid cardanol-based *meso*-tetraarylporphyrins bearing unsaturated chains were reported [15]. Metathesis reactions were then carried out on these porphyrins in order to prepare new cardanol-based products with different chemical and physical properties [15].

**Scheme 4:**
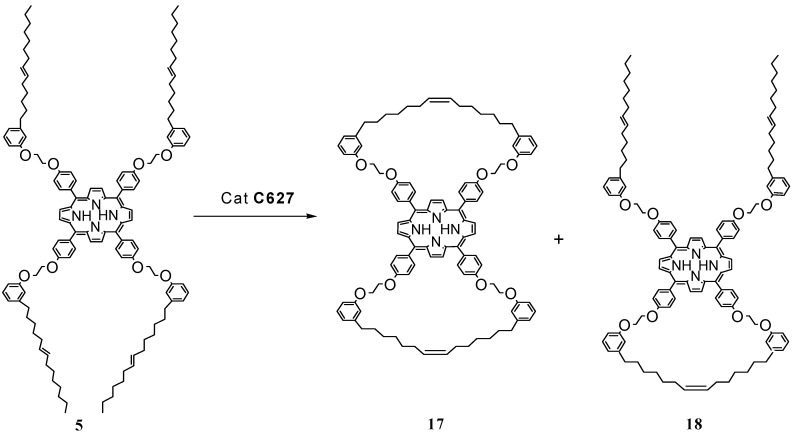
Metathesis reaction involving the cardanol based porphyrin **5**.

In 2009, new tetracardanol-based porphyrins were prepared, and an evaluation of an intra- or inter-molecular metathesis process was investigated [28]. On one of these compounds, intramolecular metathesis was performed in dichloromethane and in the presence of Ti(O*^i^*Pr)_4_ and Grubbs catalyst, involving two or all four double bonds of porphyrin’s structure (Scheme 4). Two different products were isolated, one bearing a double-bridged moiety (compound **17**), the other having two free alkyl chains (compound **18**) [16].

**Scheme 5:**
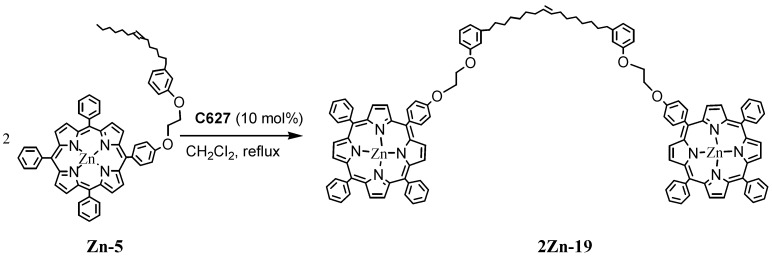
Metathesis reaction involving the cardanol-based zinc porphyrin Zn-5.

The ^1^H-NMR and LC-MS spectroscopic characterization of such compounds proved are in agreement with the proposed structure. It was possible to observe that the triplet at 0.9 ppm of the methyl protons present in **5** disappeared in **17** because of the double-bridged porphyrin-porphyrin structure (Scheme 4). In contrast, this triplet was present in the ^1^H-NMR spectrum of **18**. When the metathesis reaction was performed on the porphyrin derivative **Zn-5**, which contains only one cardanol moiety, an inter-molecular coupling occurred to produce **2Zn-19** (Scheme 5) [28]. The linking of porphyrin molecules by covalent bonds can give rise to stable, multi-porphyrin architectures and well-defined arrays, and this is a very good way for creating novel molecular wires, light-harvesting systems, energy transduction devices for molecular recognition and many other applications [29].

**Scheme 6:**
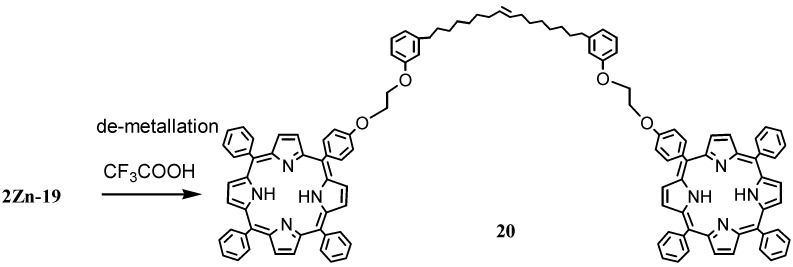
Metathesis reaction involving the cardanol based zinc porphyrin **2Zn-19**.

The route by which the zinc compound **2Zn-19** was prepared is depicted in Scheme 5. The compound **2Zn-19** was isolated in 35% yield [28]; it was successively de-metallated to obtain the non-metallic compound **20** (Scheme 6).

**Scheme 7:**
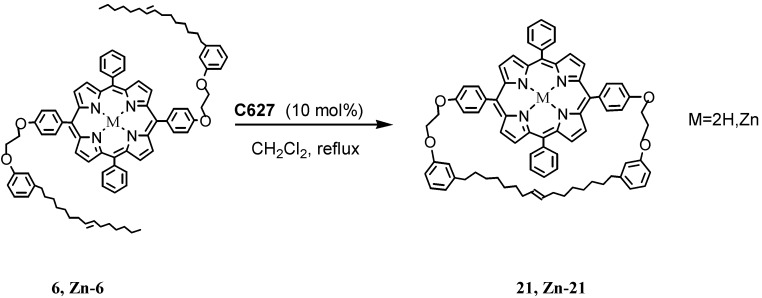
Metathesis reaction involving the cardanol based porphyrins **6** and **Zn-6**.

In the case of porphyrin derivatives with two symmetric cardanol moieties, **6** and **Zn-6**, using catalyst **C627**, produced the ring-closed products **21** and **Zn-21**, respectively in **20** and 30% yields (Scheme 7) [28].

**Scheme 8:**
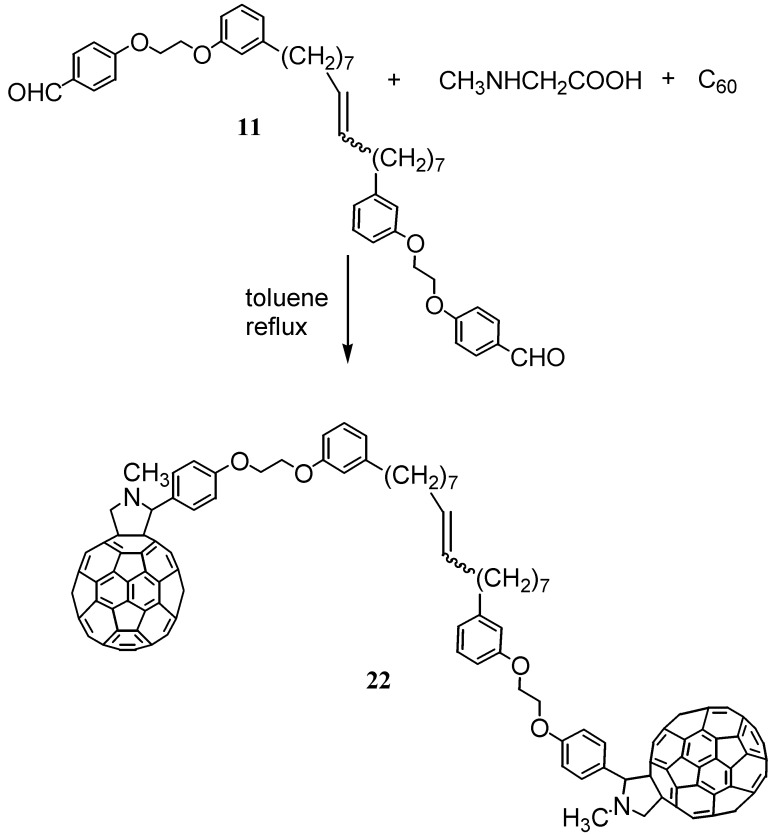
Preparation of the bis-fulleropyrrolidine **22** from the precursor **11**.

A few years ago, it was reported that new hybrid cardanol-based fulleropyrrolidines could be directly prepared via a three-component 1,3-dipolar cycloaddition reaction of fullerene (C_60_) and *N*-methyl glycine with various aldehydes in toluene. The reaction was found to proceed through the generation of azomethine ylides *in situ* [17,30]. So, in the same manner bis-fulleropyrrolidine **22** was prepared in 28% yield through a cycloaddition of the bis-azomethyne ylide in the presence of the cardanol based aldehyde precursors **11** and C_60_, as depicted in the Scheme 8.

**Scheme 9:**
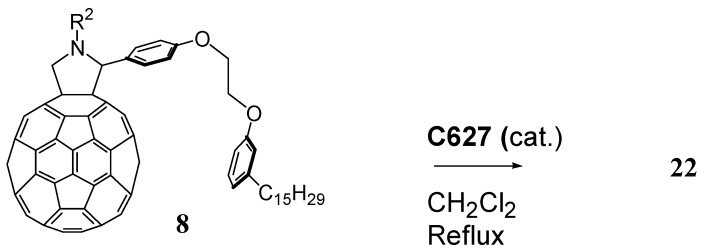
Preparation of the bis-fulleropyrrolidine **22** through homo-cross metathesis reaction of the fulleropyrrolidine **8** using **C627** as catalyst.

Alternatively, the bis-fulleropyrrolidine **22** could also be prepared according to Scheme 9.

Compound **22** was prepared in 30% yield through the metathesis reaction of the cardanol-based fulleropyrrolidine **8**, which was obtained via the cycloaddition of *N*-methylglycine in the presence of the cardanol-based aldehyde precursors **3** using C_60_. In a typical reaction, 40 mg (0.033 mmol) of fulleropyrrolidine **8** were dissolved in 21 mL of dichloromethane. Then, a solution of the catalyst **C627** (0.42 mg in 1.5 mL of CH_2_Cl_2_, 0.02 equiv.) was used to perform the metathesis reaction [17]. The crude product was purified with silica gel using toluene as the eluent.

## 5. Conclusions

Cardanol a well-known by-product of the cashew industry has been successfully used as the starting material to perform olefin metathesis reactions. The olefin metathesis reaction is particularly convenient due to commercial availablility of many α-olefins as renewable starting materials. In this paper, it has been shown that olefin metathesis reactions allow for the preparation of new cardanol-derived olefins and hybrid materials, combined with porphyrins and fullerenes, which are sometimes impossible to prepare via other methods.
